# Intravascular lymphoma of the central nervous system: a rare subtype of a common disease

**DOI:** 10.1590/0004-282X-ANP-2020-0419

**Published:** 2021-05-08

**Authors:** Ruann Melo de CARVALHO, Ana Paula Alves FONSECA, Igor Gomes PADILHA, Suellen Ka Gi MO, Flávia Fernandes Silva ZACCHI, Rita de Cássia Maciel PINCERATO, Carlos Sérgio CHIATTONE

**Affiliations:** 1 Hospital Samaritano Higienópolis, UnitedHealth Group, Departamento de Neurorradiologia, São Paulo SP, Brazil. Hospital Samaritano Higienópolis UnitedHealth Group Departamento de Neurorradiologia São Paulo SP Brazil; 2 Hospital Samaritano Higienópolis, UnitedHealth Group, Departamento de Hematologia, São Paulo SP, Brazil. Hospital Samaritano Higienópolis UnitedHealth Group Departamento de Hematologia São Paulo SP Brazil; 3 Grupo Fleury, Departamento de Anatomia Patológica, São Paulo SP, Brazil. Grupo Fleury Departamento de Anatomia Patológica São Paulo SP Brazil

**Figure 1.**
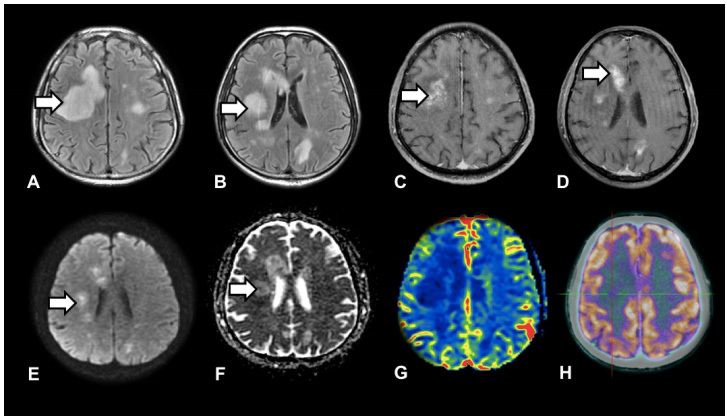
Magnetic resonance imaging findings of intravascular lymphoma. (A and B) Fluid-attenuated inversion recovery axial images showed diffuse multiple hyperintensities of the cerebral white matter. (C and D) The perivascular curvilinear enhancement on T1-weighted imaging with gadolinium expanded markedly. (E and F) Diffusion-weighted imaging and apparent diffusion coefficient map showed restricted diffusion. (G and H) Perfusion magnetic resonance imaging (relative cerebral blood volume) and positron emission tomography - computed tomography were practically unremarkable.

**Figure 2.**
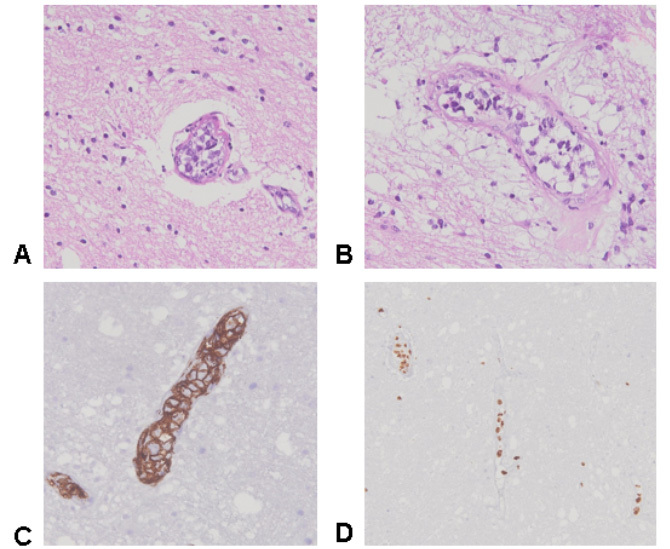
Brain pathology showing small blood vessels filled with lymphoma cells and perivascular reactive lymphocytes (A and B). Immuno his to chemical staining showing CD20+ (C and D).

A 67-year-old man had a sudden onset of headache and aphasia, evolving to spontaneous improvement. A few months later, he developed dysarthria and left hemiparesis. Brain MRI showed several punctate lesions with perilesional edema, perivascular enhancement and restricted diffusion on DWI (Figure 1). PET-CT demonstrated mild uptake (Figure 1). Anatomopathological and immunohistochemical analysis were compatible with intravascular large B-cell lymphoma (IVL) (Figure 2). Treatment with R-CHOP and intrathecal methotrexate was established, with favorable response due to high tumor sensitivity^1^. Approximately half of IVL cases are diagnosed only after autopsy^2^. The main differential diagnoses are vasculitis, neurosarcoidosis, and ischemic stroke^3^^,^^4^.
